# Does lateral arm technique decrease the rate of clip migration in stereotactic and tomosynthesis-guided biopsies?

**DOI:** 10.1186/s13244-021-01136-w

**Published:** 2021-12-20

**Authors:** Olena Weaver, Ethan O. Cohen, Rachel E. Perry, Hilda H. Tso, Kanchan Phalak, Ashmitha Srinivasan, Roland Bassett, Jessica W. T. Leung

**Affiliations:** 1 Department of Breast Imaging, Unit 1350, The University of Texas MD Anderson Cancer Center, 1515 Holcombe Boulevard, Houston, TX 77030 USA; 2 Synergy Radiology Associates, 7026 Old Katy Rd, Ste. 276, Houston, TX 77024 USA

**Keywords:** Stereotactic breast biopsy, Lateral arm approach, Mammography, Clip migration, Hematoma

## Abstract

**Background:**

Mammography-guided vacuum-assisted biopsies (MGVAB) can be done with stereotaxis or digital breast tomosynthesis guidance. Both methods can be performed with a conventional (CBA) or a lateral arm biopsy approach (LABA). Marker clip migration is relatively frequent in MGVAB (up to 44%), which in cases requiring surgery carries a risk of positive margins and re-excision. We aimed to compare the rates of clip migration and hematoma formation between the CBA and LABA techniques of prone MGVAB. Our HIPAA compliant retrospective study included all consecutive prone MGVAB performed in a single institution over a 20-month period. The LABA approach was used with DBT guidance; CBA utilized DBT or stereotactic guidance. The tissue sampling techniques were otherwise identical.

**Results:**

After exclusion, 389 biopsies on 356 patients were analyzed. LABA was done in 97 (25%), and CBA in 292 (75%) cases. There was no statistical difference in clip migration rate with either 1 cm or 2 cm distance cut-off [15% for CBA and 10% for LABA for 1 cm threshold (*p* = 0.31); 5.8% or CBA and 3.1% or LABA for 2 cm threshold (*p* = 0.43)]. There was no difference in the rate of hematoma formation (57.5% in CDB and 50.5% in LABA, *p* = 0.24). The rates of technical failure were similar for both techniques (1.7% for CBA and 3% for LABA) with a combined failure rate of 1%.

**Conclusions:**

LABA and CBA had no statistical difference in clip migration or hematoma formation rates. Both techniques had similar success rates and may be helpful in different clinical situations.

## Keypoints

- Stereotactic breast biopsies can be performed with conventional (CBA) or “lateral arm” (LABA) approach.
- LABA does not decrease the rate of clip migration.
- LABA is not associated with increased rate of hematoma formation.
- LABA is complementary to CBA for some clinical applications.
- Availability of both techniques increases the technical success rate of biopsies to 99%.

## Background

Stereotactic biopsy (SB) is a safe and effective method of sampling suspicious mammography (MG) detected non-palpable imaging findings that are not visible on ultrasound [1–4]. It has become the standard of care for MG-only detected lesions, replacing surgical excisional biopsy [1, 5, 6]. SB is most often performed for microcalcifications, which may be the earliest sign of breast cancer, but are not readily detectable by any other breast imaging modality [2, 5, 7].

In recent years, digital breast tomosynthesis (DBT)-guided biopsy (TB) has been introduced as an alternative method to SB. TB produces similar or superior technical success compared to SB, especially for low-contrast non-calcified lesions. This is possible due to the direct lesion depth determination, which does not depend on a triangulation process requiring visualization of the target on both stereotactic projections 30 degrees apart from each other [8–12].

**Fig. 1:**
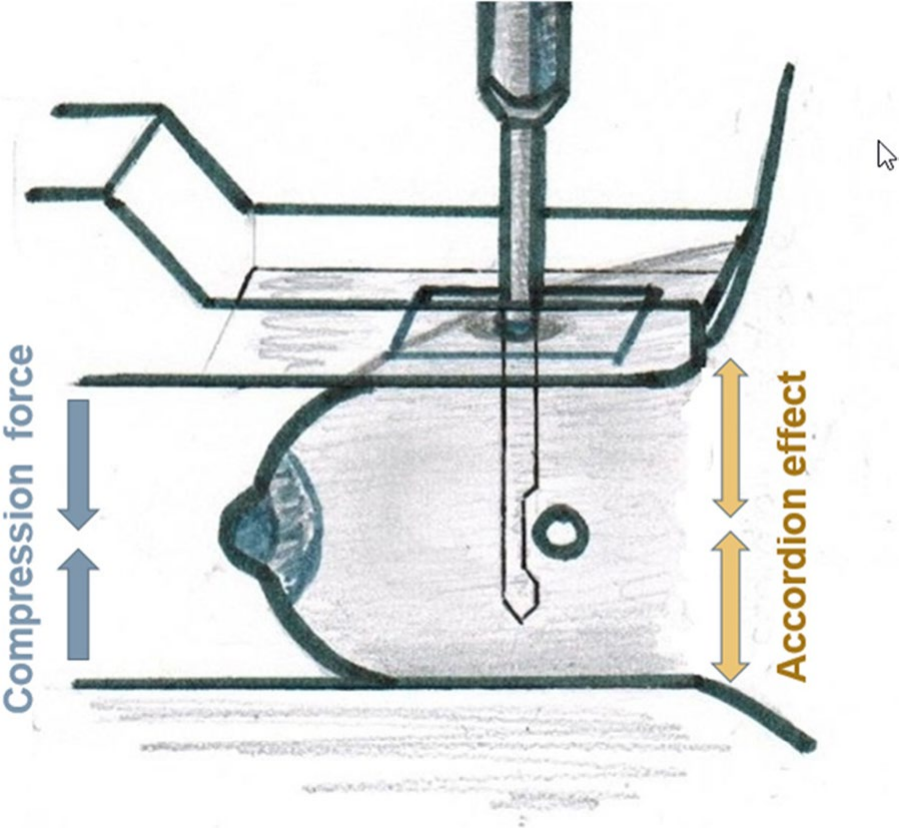
Conventional biopsy approach demonstrating the needle position orthogonal to the compression plate, and parallel to the compression force. The needle position is also in the direction of the accordion effect, thought to contribute to clip migration

**Fig. 2:**
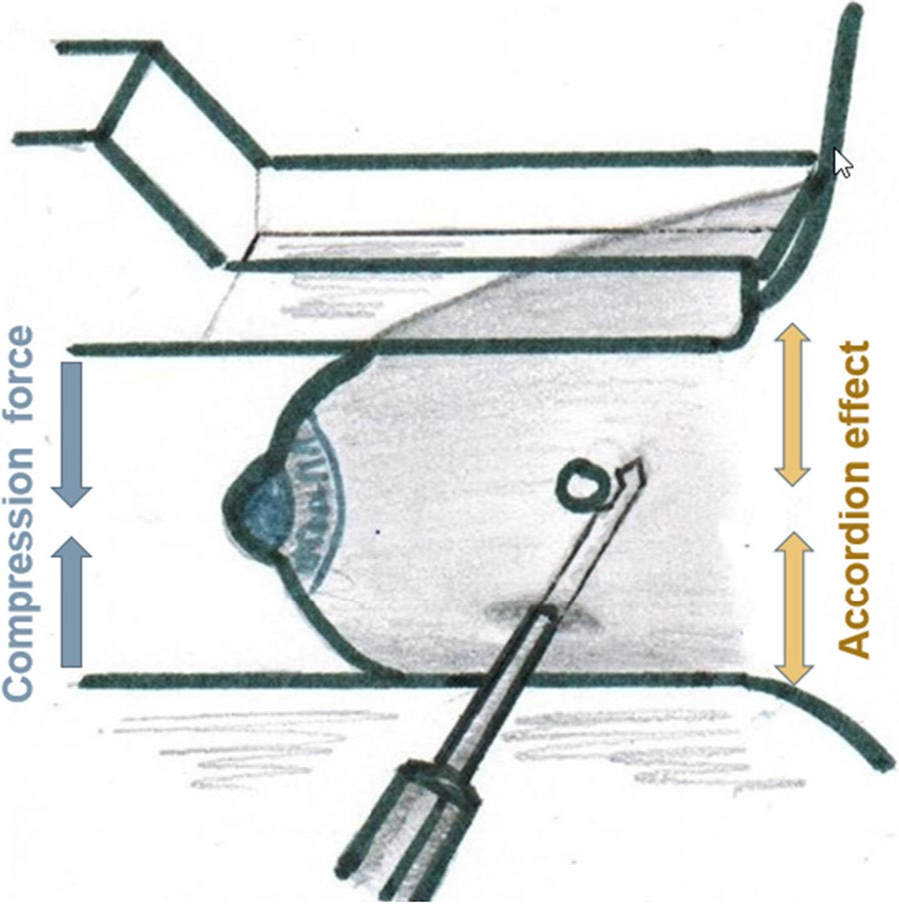
Lateral arm biopsy approach demonstrating a needle position parallel to the compression plate. It is orthogonal to the compression force and to the direction of the accordion effect (which is thought to contribute to clip migration). Because of this orthogonal relationship, it was hypothesized that migration rate and distance would be reduced

Two approaches are proposed for MGVAB with either SB and DBT-guided techniques: the conventional (vertical/orthogonal) biopsy approach (CBA), when the needle is advanced perpendicularly to the compression plate, in the direction of maximal tissue compression (Fig. 1); and the lateral arm biopsy approach (LABA), when the needle is advanced parallel to the compression plate, in the direction orthogonal to the maximal compression [13] (Fig. 2). LABA is mostly advocated for thin breasts and peripheral lesions, and for decreasing the masking effect of lidocaine on subtle mammographic findings [3, 13–15].

Placing a marker clip after MGVAB has become a standard method of localizing the biopsy site, especially if the original target is small and is likely to be completely removed with the biopsy [16–19]. The precise placement of the marker is crucial for possible subsequent surgical excision, when the original target is no longer visible [18–20]. Clip malpositioning or migration may result in positive surgical margins and a greater re-excision rate [2, 20, 21]. At the same time, the clip migration rate is relatively high, ranging from 2 to 44% across all methods and clip types, when using a threshold of 1 cm (as recommended by the British National Health Service Breast Screening Program quality assurance guidelines for surgeons, and as preferred by the surgeons at our institution [22]). The range of clip migration is reported to be 7–22% at a threshold of 2 cm [16, 18, 21, 23–25].

With CBA, clip migration happens in the orthogonal direction (z-axis), which is perpendicular to the plane of breast compression [17, 24]. Thin breast compression, fatty breast tissue composition, posterior lesion size, and a large number of core samples were associated with clip migration in clinical studies [26]. One of the proposed mechanisms for clip migration is the “accordion effect”, when the breast tissue compressed for biopsy is allowed to re-expand post-biopsy, forcing the clip to move along the z-axis away from the original target during the re-expansion process [17, 18, 27] (Fig. 1). Considering the principle of LABA, it appears that a lower clip migration rate may be one of its benefits. If the biopsy needle is inserted in the direction of maximal tissue expansion, the described “accordion effect” could be minimized, resulting in a more precise position of the marker clip [17, 23]. We utilized both CBA and LABA and compared the clip migration rates for these two approaches.

## Materials and methods

### Study population

We conducted a Health Insurance Portability and Accountability Act-compliant retrospective data analysis of all MGVAB (SB and TB) performed at a single institution over 20 months. The biopsies were performed within a quality improvement (QI) project initiated after installation of a new Affirm prone 2D/3D stereotactic biopsy system (Hologic, Inc., Marlborough, MA) in August 2016 and included all consecutive female patients who required a MGVAB as a standard of care. The purpose of the QI project was implementation of both conventional and lateral arm approaches and optimization of the stereotactic technique. A waiver of informed consent was obtained from the institutional review board for this retrospective review.

### Biopsy techniques

Six fellowship trained breast radiologists with 3–10 years of experience performed CBA and LABA without randomization. None of the radiologists had previous experience with the LABA technique, but all underwent training by the manufacturer.

**Fig. 3:**
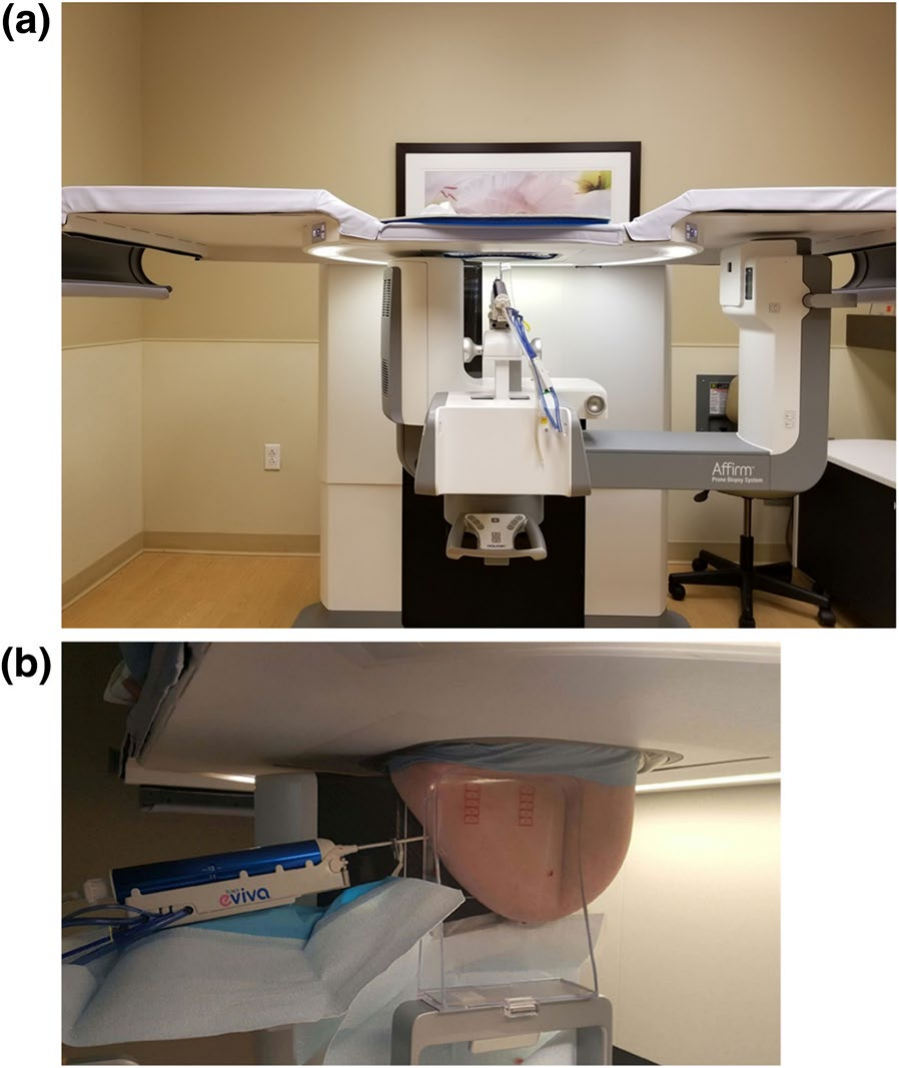
**a** Photograph of the lateral arm biopsy approach set-up. Affirm prone 2D/3D stereotactic biopsy system (Hologic, Inc.) with a 9-gauge Eviva needle (Hologic, Inc.) in place. **b** Photograph of the lateral arm biopsy approach set-up. 9-gauge Eviva needle (Hologic, Inc.) in post-fire position is inserted in the direction parallel to the compression plate

The biopsies with CBA were performed with stereotaxis or DBT guidance, using the standard manufacturer-recommended techniques described in detail elsewhere [1, 3, 10, 12, 28]. The biopsies with LABA were performed using DBT targeting only by agreement among radiologists (Fig. 3A, B). The choice of the CBA or LABA technique was made at discretion of the radiologist performing the procedure and was not limited by the usual indications for the LBA technique such as thin breast or a superficial location of the target. All biopsies were performed using a 9-gauge vacuum-assisted core biopsy system (Eviva; Hologic, Inc.). A marker clip was placed at the end of every biopsy. TriMark or SecurMark clips (Hologic, Inc.) were used for this purpose, based on radiologist preference and the presence (and type) of other clip(s) in the breast. In both CBA and LABA, paddle compression was released after confirmation of clip deployment via post-clip placement imaging, while the patient was still in the biopsy position. The paddle compression release was performed in the same fashion in both CBA and LABA. Immediately after the needle was removed and paddle compression released, manual compression was applied to the biopsy sites in the direction of needle insertion for both CDA and LABA techniques.

### Lateral arm biopsy approach

Due to the fact that LABA was a new technique for all radiologists, it was standardized for the QI project as follows:

1. DBT scout imaging was used for targeting.
2. The target was chosen 3–5 mm below the lesion in the DBT slice best demonstrating the center or the bulk of the lesion.
3. The needle trough was kept at 12:00 position for best visualization of the lesion above the needle.
4. DBT pre-fire images were obtained to make sure that the target and the needle tip were in the same plane.
5. A single 2D post-fire image was obtained with the trough of the biopsy needle at 12:00 position with the goal of visualizing the target immediately above the open trough. If in doubt, DBT images were obtained to see if the target was off center in relation to the needle.
6. Sampling was done preferentially in the direction of the target, and not around the clock.
7. A single 2D post-clip image was obtained.
8. After the procedure, release of compression and removal of the needle were done at the same time, in an attempt to “lock” the clip in place. Once the needle was removed and the compression released, focal pressure was applied to minimize bleeding.

### Evaluation of clip migration

Clip migration was evaluated on post-procedure mammograms by measuring the distance from the center of the original target to the clip on 2 projections (craniocaudal [CC] and lateromedial [LM]) mammograms, and recording the largest distance. When the entire lesion was removed, the measurements were done from the estimated center of the target using mammographic tissue landmarks or a post-procedure hematoma or air. Two potentially clinically relevant migration cut-offs were applied: distance ≤ 1 cm or distance ≤ 2 cm.

### Evaluation of hematoma formation

The presence and size of hematomas were evaluated on immediate post-procedure 2D mammograms in CC and LM projections. If a discernable hematoma was present, it was measured in 3 dimensions, and the largest measurement was recorded for analysis. For the calculation of the rate of hematoma formation, a maximal hematoma diameter of 1 cm was considered the threshold for recording the case as positive for hematoma formation.

### Pathology

Pathology results were obtained from the pathology reports in patients’ medical records. The results were grouped as benign; malignant (invasive ductal carcinoma, invasive lobular carcinoma, ductal carcinoma in situ); and high risk (atypical ductal hyperplasia, atypical lobular hyperplasia, lobular carcinoma in situ, flat epithelial atypia, or any combination of benign lesions with atypical features).

### Data review

The data pertinent to the biopsy technique were recorded in real time by radiologists and technologists in data logs designed for quality improvement purposes. The images were reviewed on SecurView workstations (Hologic; Inc.) or a radiology PACS system (Centricity; GE). All numeric data were compiled and summarized in an Excel spreadsheet for analysis.

### Statistical analysis

Our primary interest was to compare biopsies performed using the conventional technique to those performed using the lateral technique. Wilcoxon rank-sum tests were used to compare the distribution of continuous variables between groups. Fisher’s exact tests were used to compare the distribution of categorical variables between groups. All statistical analyses were performed using R version 3.6.1 software. All statistical tests used a significance level of 5%. No adjustments for multiple testing were made.

## Results

390 biopsies were performed for 357 patients. One patient had a single site biopsy that failed both CAB and LABA techniques due to non-visualization of the target, and was excluded from the group analysis, resulting in 389 biopsies for 356 patients (Table 1). LABA was done in 97 (25%), and CBA in 292 (75%) cases. Patient age ranged from 32 to 86 years (median 55). The LABA and CBA groups were not statistically different in patient age (median 55, SD 10.8 and 10.1, respectively) (Table 2), or in PPV3 for invasive or in-situ cancer (24% and 21%, respectively, *p* = 0.18) (Table 3).

**Table 1:** Nine cases with failed biopsy attempts with initial technique

Cases and their characteristics	Initial technique	Back-up technique	Result
Limited visualization of amorphous microcalcifications	**LABA**	**CBA**	**Failure**
Subcutaneous microcalcifications	**Not amenable to CBA**	*LABA*	*Success*
Subcutaneous microcalcifications	**Not amenable to CBA**	*LABA*	*Success*
Multiple superficial blood vessels	**LABA**	*CBA*	*Success*
Thin breast with tissue rotation/displacement by the needle	**LABA**	*CBA*	*Success*
Posterior and lateral location of the target	**LABA**	*CBA*	*Success*
Non-visualization of microcalcifications	**CBA**	N/A	**Failure**
Non-visualization of microcalcifications	**CBA**	N/A	**Failure**
Non-visualization of microcalcifications	**CBA**	N/A	**Failure**

Bold denotes failure, italics denotes success. Three CBA cases were not converted to LABA and remained categorized as failure

**Table 2:** Patient characteristics (continuous) by technique

Variable	Group	*N*	Min	*q*1	Med	Mean	*q*3	Max	SD
Age	CBA	265	33	48	55	55.600	62	86	10.056
	LABA	91	32	48	55	55.824	65	82	10.800
*p* = 0*.*77	All	356	32	48	55	55.657	63	86	10.236

*N* number of patients

**Table 3:** Pathology results for CBA and LABA biopsies

Pathology	nCBA	%CBA	nLABA	%LABA	*n*_all_	%all
Benign	196	67.1	58	59.8	254	65.3
DCIS	49	16.8	17	17.5	66	17.0
High risk	35	12.0	16	16.5	51	13.1
IDC	10	3.4	3	3.1	13	3.3
IDC + DCIS	2	0.7	1	1.0	3	0.8
ILC	0	0.0	2	2.1	2	0.5
All	292	100.0	97	100.0	389	100.0

High-risk pathology includes atypical ductal hyperplasia, atypical lobular hyperplasia, lobular carcinoma in situ, flat epithelial atypia, and any benign lesion with atypical features

*DCIS* ductal carcinoma in situ, *IDC* invasive ductal carcinoma, *ILC* invasive lobular carcinoma

There was no statistical difference in clip migration rate with either a 1 cm or 2 cm migration distance cut-off. For a 1 cm cut-off, the rate of clip migration was 15% for CBA and 10% for LABA (*p* = 0.31). For a 2 cm cut-off, the rates were 5.8% for CBA and 3.1% for LABA (*p* = 0.43) (Table 4). The distance of clip migration ranged from 0 to 6.6 cm for CBA (mean 0.48 cm, SD = 0.98); in LABA it ranged from 0 to 3.4 cm (mean 0.34, SD = 0.62), with no significant difference between the two techniques (Tables 4, 5).

**Table 4:** Biopsy characteristics (categorical) by technique

Variable	Threshold value	*N* CBA	% CBA	*N* LABA	%LABA	*N* All	% All
Clip migration	≤ 1	249	85.3	87	89.7	336	86.4
Distance > 1	> 1	43	14.7	10	10.3	53	13.6
*p* = 0*.*31	All	292	100.0	97	100.0	389	100.0
Clip migration	≤ 2	275	94.2	94	96.9	369	94.9
Distance > 2	> 2	17	5.8	3	3.1	20	5.1
*p* = 0*.*43	All	292	100.0	97	100.0	389	100.0
Hematoma	No	168	57.5	49	50.5	217	55.8
	Yes	124	42.5	48	49.5	172	44.2
*p* = 0*.*24	all	292	100.0	97	100.0	389	100.0

**Table 5:** Biopsy characteristics (continuous) by technique

Variable	Group	*N*	Min	*q*1	Mean	*q*3	Max	SD
Clip migration	CBA	292	0	0.000	0.482	0.800	6.600	0.977
Distance	LABA	97	0	0.000	0.341	0.600	3.400	0.624
*p* = 0*.*73	All	389	0	0.000	0.447	0.700	6.600	0.903
Hematoma	CBA	127	0	1.200	1.573	1.800	5.200	0.678
Size	LABA	48	0	1.200	1.502	1.725	2.800	0.534
*p* = 0*.*69	All	175	0	1.200	1.554	1.800	5.200	0.641

The rate of hematoma formation was similar for both techniques (57.5% in CBA and 50.5% in LABA, *p* = 0.24) (Table 2). The sizes of associated hematomas ranged from 0 to 5.2 cm in CBA (mean 1.57, SD = 0.68) and from 0 to 2.8 cm in LABA (mean 1.5, SD = 0.53) (Tables 4, 5).

**Fig. 4:**
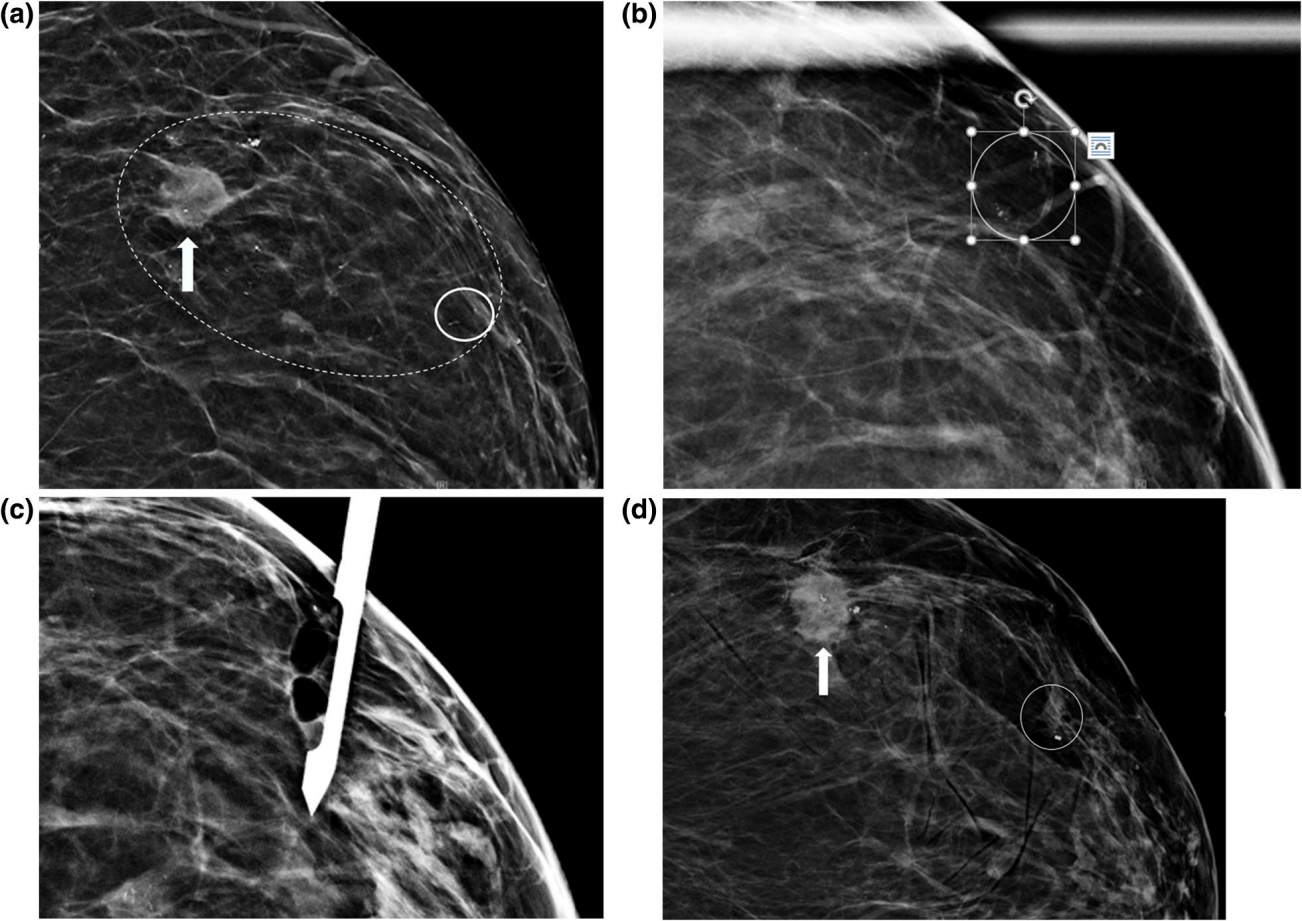
**a** Grouped microcalcifications not considered amenable to CBA, but successfully biopsied with LABA. Craniocaudal mammogram of a 58 y.o. woman with a highly suspicious 2 cm mass in the left breast (arrow) with an associated 10 cm area of fine pleomorphic microcalcifications in segmental distribution (dashed ellipse). The most anterior extent of the calcifications (solid circle) was recommended for a stereotactic biopsy. **b** Grouped microcalcifications (circle) not considered amenable to CBA, but successfully biopsied with LABA. CC compression magnification view of the left breast demonstrating the microcalcifications in question in a superficial position. **c** Grouped microcalcifications not considered amenable to CBA, but successfully biopsied with LABA. An intra-procedure post-sample CC mammogram demonstrates the biopsy needle parallel to the compression plate, with the trough situated just beyond the skin and with all microcalcifications removed. **d** Grouped microcalcifications not considered amenable to CBA, but successfully biopsied with LABA. Post-procedure CC mammogram demonstrating a stereotactic biopsy clip in appropriate position, with a minimal hematoma and no sign of clip migration (circle). Pathology demonstrated grade 2 DCIS. The index mass was biopsied with ultrasound guidance, demonstrating invasive ductal carcinoma, grade 2. A ribbon-shaped clip was placed (arrow)

The overall combined biopsy failure rate was 1% (4 of 390 targets). Both techniques failed in one case, and this case was excluded from the subgroup analysis. Individual failure rates were similar for each needle approach in the subgroup analysis. CBA failed in 5 cases (1.7%): 2 biopsies of the superficial lesions were enabled only by the presence of LABA, and would otherwise have been cancelled and referred for a wire localized surgical excisional biopsy (Fig. 4A–D). Three CBA failed to retrieve calcifications (no conversion to LASB was made). Attempted LABA had to be converted to CBA in 3 cases (3%) for the following reasons: (1) thin breast with heterogeneously dense composition, which caused tissue rotation/displacement by the needle and resulting non-visualization of calcifications; (2) multiple vessels in the presumed course of the needle, which could not be avoided with changed LABA positioning, but were eliminated with CBA; 3) posterior and lateral location of the target.

## Discussion

Despite our initial hypothesis that LABA may decrease the rate or the distance of clip migration after MGVAB by eliminating the accordion effect thought to contribute to clip migration, this study demonstrates no statistical difference in the rate of clip migration between the two approaches. This remained true when suggested clinically significant cut-offs of either 1 or 2 cm were used. Therefore, a small lesion with potential of complete removal should not on its own influence the choice of biopsy technique. However, as previously reported, and as confirmed in our study, different approaches may be chosen depending on other target and breast-related considerations, such as thin breast or superficial lesion location. In our study, the benefit of the lateral arm technique was most obvious in 2 biopsies of superficial lesions, when biopsy would not have been possible if only CAB was used. The success was enabled by a direct visualization of the needle trough and its position in relation to the overlying skin, and the possibility of precisely controlling this position. On the other hand, in our study the lateral arm technique could not be performed in a patient with multiple superficial blood vessels. This occurred because there is limited flexibility in tissue rotation and the potential needle site entry area in LABA approach, and the tightly arranged vessels were further compressed together in the potential course of the needle, eliminating a window for a safe needle insertion. CAB technique in this case allowed spreading of the vessels with a larger window for the biopsy. Contrary to the reported superiority on LABA in thin breasts, in our study the presence of a thin breast with dense tissue composition prevented LABA in one case. This appeared to happen because there is no firm tissue stabilization on the side opposite to needle insertion in LABA. In this case of a woman with dense tissue, the advancing needle rotated the tissues and deviated, displacing and obscuring the target. This can potentially be remediated by applying manual counter-pressure on the side of the breast opposite to the needle insertion.

The lack of significant preference for LABA in our group (LABA was chosen by the radiologists in 25% of cases) may stem from a generally high comfort level of the radiologists with performing technically challenging biopsies with additional manipulations, such as utilization of petite size needles, cushioning, and skin elevation, which are described in detail elsewhere [1]. This familiarity with the traditional techniques may have caused some hesitancy on the part of radiologists to attempt LABA after a failed CBA, even when it may have been helpful.

Our study had several limitations. Because LABA was new for all radiologists, this could have affected both the choice of technique and the performance of biopsies due to a learning curve. In addition, we did not take into account the difference between marker types. We used both SecurMark clips with bio-absorbable nets and net-free TriMark clips. The choice of a biopsy clip was random in most cases and was not accounted for in our study. The presence of a bio-absorbable net may have affected clip migration rates. Moreover, we did not systematically record the paddle compression force to evaluate whether this could be a confounding factor in clip migration.

## Conclusions

Our study did not demonstrate a significant reduction in clip migration rate with LABA compared to CBA, contrary to our initial hypothesis. Rather, both techniques proved to be useful and complementary for different clinical scenarios. In our study, LABA was particularly helpful for superficial lesions. The choice of a particular technique should be based on the target and breast characteristics. Having access to both LABA and CBA allows successful tissue sampling in 99% of cases.

## Data Availability

All data generated or analyzed during this study are included in this published article.

## Abbreviations

CBA: Conventional biopsy approach
DBT: Digital breast tomosynthesis
LABA: Lateral arm biopsy approach
MG: Mammography
MGVAB: Mammography-guided vacuum-assisted biopsies
